# ACSS2-related autophagy has a dual impact on memory

**DOI:** 10.1186/s41016-019-0162-y

**Published:** 2019-06-11

**Authors:** Hao Zhang, Zujian Xiong, Qin He, Fan Fan

**Affiliations:** Central South University, Xiangya Hospital, Changsha city, Hunan province China

**Keywords:** ACSS2, TFEB, Memory, Histone acetylation

## Abstract

Autophagy is an intracellular degenerative pathway which is responsible for neuronal survival. Under the condition of nutrient deprivation, autophagy can lead to dysfunction in memory consolidation. AMPK/mTOR pathway is currently the most studied autophagy mechanism, while recently researchers have proved ACSS2 can also affect autophagy. ACSS2 is phosphorylated at Ser659 by AMPK and then forms a translocation complex with Importin α5 to translocate into the nucleus. This process interacts with TFEB, resulting in upregulated expression of lysosomal and autophagosomal genes. These upregulations inhibit synaptic plasticity and hence memory functions. On the other hand, ACSS2 is also recognized as a regulator of histone acetylation. After recruiting CBP/p300 and activating CBP’s HAT activity in the nucleus, ACSS2 maintains the level of localized histone acetylation by recapturing acetate from histone deacetylation to reform acetyl-CoA, providing substrates for HAT. The increase of histone acetylation locally enhanced immediate early gene transcription, including *Egr2*, *Fos*, *Nr2f2*, *Sgk1*, and *Arc*, to benefit neuronal plasticity and memory in many ways.

## Background

Autophagy is a natural, regulated mechanism in cells that disassembles unnecessary or dysfunctional component for survival under starvation conditions. In the past, researches revealed two classic pathways for autophagy. In the classic autophagy pathway, mTOR, inhibited by 5′ AMP-activated protein kinase (AMPK), is a major inhibitor of unc-51-like kinase 1 (ULK1) which contributes to autophagy [1]. In the other classic autophagy pathway, Beclin1, inhibited by B cell lymphoma 2 (Bcl-2), forms a complex with class III PI-3 kinases, which promotes autophagy [2]. In most eukaryotes, autophagy depends on cellular nutrient status. When cells are starving, autophagy will be essential for cellular survival [3, 4].

Few reports relate autophagy with synaptic remodeling, phosphatidylinositol 3-kinase (PI3K)-mTOR pathway, or endosome-dependent proteolysis. In fact, abnormal autophagic vesicles disrupt presynaptic terminals and cause axonal dystrophy [5]. Also, researchers have reported the roles of autophagy in synaptic plasticity, dendritic neurons [6], and cultured neurons. Connections between memory and synaptic plasticity were reported in several researches [7, 8]. Associated with lysosomal and autophagosomal genes, acetyl-CoA synthetase 2 (ACSS2), a metabolic enzyme, was reported to relate with memory functions. Knock-in of ACSS2 mutants that are inactive in glioblastoma cells eliminates glucose deprivation-induced lysosomal biogenesis and autophagy, which in turn reduce cell survival [9]. AMPK-dependent ACSS2 Ser659 phosphorylation and the subsequent binding of ACSS2 to Importin α5 mediates the nuclear translocation of ACSS2. This translocation is induced by energy stress [10, 11]. In the nucleus, at first, ACSS2 binds to transcription factor EB (TFEB) and utilizes the acetate generated from histone deacetylation; then, ACSS2 locally produces acetyl-CoA for histone acetylation in the promoter regions of TFEB target genes. Acetyl-CoA produced this way might participate in histone acetylation process.

As known, long-term memory formation relies on the epigenetic regulation of genes. Among epigenetic regulations, histone acetylation has been one of the most studied and best characterized under both physiological and pathological conditions [12, 13]. When regulating autophagy by activating autophagy-related gene expression, ACSS2 is likely to promote histone acetylation at the same time and consequently benefit memory formation. Therefore, ACSS2 involved in autophagy process may have a dual impact on memory consolidation.

## AMPK induces ACSS2 to translocate into the nucleus

**Fig. 1:**
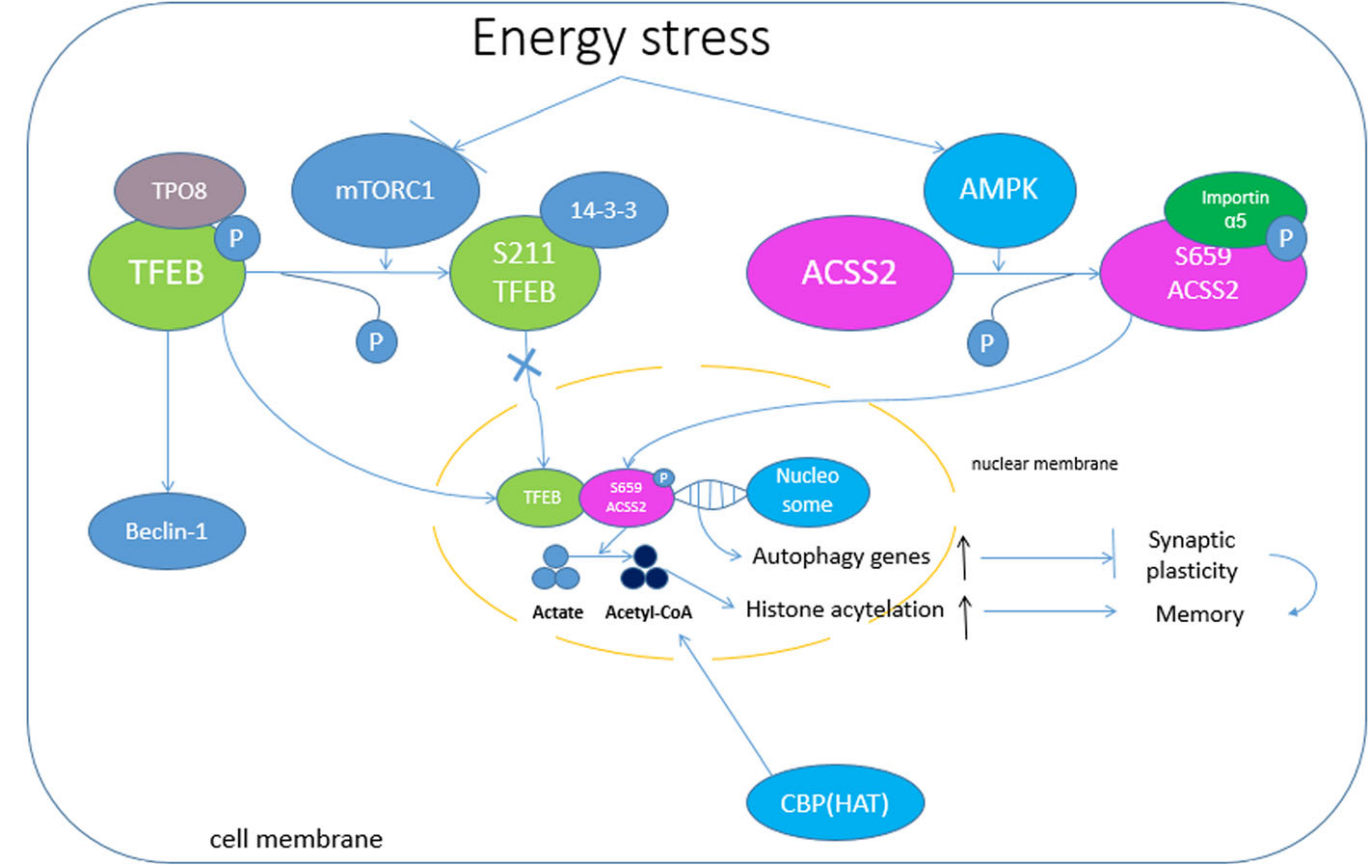
Glucose deprivation resulted in AMPK-phosphorylation-dependent formation of an ACSS2 and TFEB complex in the promoter regions of lysosomal and autophagy genes, where ACSS2 incorporated acetate from the turnover of histone acetylation into acetyl-CoA for histone H3 acetylation, gene expression, and enhanced lysosomal biogenesis and autophagy

AMPK is a key regulator of glucose and lipid metabolism corresponding with alteration of nutrient and energy [14]. The heterotrimeric protein is formed by α, β, and γ subunits, while α subunit has two isoforms: α1 and α2. AMPK is a sensor of cellular energy stress and an initiator of the autophagy pathway. In ACSS2 metabolism, AMPK was reported to relay ACSS2 S659 phosphorylation [9], which results in exposing the ACSS2 nuclear localization signal (NLS), causing ACSS2 to bind with Importin α5 and to pass through the nucleus for further transduction (Fig. 1). Li [9] proved that AMPK inhibitor compound C blocks glucose deprivation-induced nuclear translocation of ACSS2. Further, ACSS2 fails to translocate into the nucleus with AMPK α1 or α2 subunit deficiency. AMPK upstream regulators are Ca2+/calmodulin-dependent protein kinase kinase b (CaMKKb) and liver kinase B1 (LKB1), which activate APMK by phosphorylating AMPK at T172, favoring its direct binding with ACSS2. Under nutrient deprivation, ATP to ADP ratio decreases, and AMP to ATP ratio increases markedly through adenylate kinase amplification, promoting physical association of AMP and γ subunit of AMPK. The connection contributes to AMPK phosphorylation by LKB1 [9], inhibiting dephosphorylation by protein phosphatases. These results indicate that the activation of AMPK can be influenced by AMP concentration and AMP to ATP ratio. Li [9] revealed that AMPK activates ACSS2 upon S659, one of the evolutionarily conserved residues of ACSS2. Mutation of ACSS2 S659 into Alanine (S659A) could abrogate AMPK-mediated ACSS2 phosphorylation, ensuing nuclear translocation, as detected by antibody technique, immunofluorescent, and immunoblot analysis. ACSS2 S659 phosphorylation exposes NLS of ACSS2 to bind Importin α5. Importin α5 then links ACSS2 with importin β, which in turn docks the ternary complex at the nuclear pore to facilitate their endonuclear translocation. Functional studies and immunoprecipitation confirmed the role that AMPK-mediated ACSS2 S659 phosphorylation plays in binding ACSS2 to importin α5.

## TFEB-associated ACSS2 upregulates lysosomal and autophagy gene expression in the nucleus

In the process of lysosomal genesis, TFEB functions as a master gene that coordinates expression of lysosomal hydrolases and membrane proteins in autophagy [15]. Extracellular nutrients’ level adjusts the TFEB activity; meanwhile, extracellular signal-regulated kinase 2 mediates serine phosphorylation, regulating nuclear localization and activity of TFEB [16]. TFEB promotes autophagy by enhancing the expression of beclin-1, a specific gene for mammalian involvement in autophagy, and consequently increases cell proliferation and cell survival. Palmieri et al. [17] found that beclin-1 was one of the direct goals of TFEB. It was reported that TFEB activation also promotes expression of other autophagy genes in the condition of glucose deprivation [18, 19], including lysosomal enzymes cathepsin A (encoded by *CTSA*) and the lysosomal membrane protein LAMP1 (encoded by *LAMP1*). Glucose deprivation induces autophagy and lysosomal organisms, which is the response of cell survival to metabolic stress. Besides, TFEB translocates into the nucleus with the help of Importin 8 (IPO8), a specific protein for TFEB nuclear translocation. IPO8 disassociates with TFEB immediately in the nucleus, and TFEB combines with ACSS2 that is phosphorylated at S659 [9] (Fig. 1). Translocation of ACSS2 into the nucleus is an essential prerequisite of TFEB activation [9]. Immunoblotting analysis shows that IPO8 has greater ability to bind to TFEB than does ACSS2 R664/665A [7], and depletion of IPO8 [18] enabled wild-type (WT) TFEB to bind to ACSS2 R664/665A in response to glucose deprivation. TFEB, in complex with nuclear ACSS2, binds to the promoter regions of lysosomal and autophagosomal genes so as to promote the expression of these genes [9]. The analysis of immune co-precipitation [9] showed that in U87 cells, TFEB and ACSS2 were incorporated in the nucleus, rather than in cell cytosol. In addition, the joint immunological precipitation analysis [9] failed to find the complex formation of ACSS2 and other TFE family members TFE3, TFEC, or MITF [18], suggesting that ACSS2 binds with TFEB specifically. TFEB and ACSS2 complex interact with promoter regions of lysosomal genes. In addition, Li [9] came to a conclusion that glucose deprivation induces mRNA and protein expression of these lysosomal genes, such as MAP1LC3B, ATG3, and WIPI-1. These inductions were largely eliminated by knock-in of ACSS2 mutants (S659A, r664/665a, T363K), which suggests that autophagy induced by glucose deprivation may be associated with intact structures as well as ACSS2 function. Moreover, the lysosomal inhibitor chloroquine was found no effect on the inhibited LC3B expression mediated by ACSS2 R664/665A [9]. Thus, the autophagosomal gene transcriptional regulation of glucose deprivation, depending on nuclear ACSS2/TFEB, is not caused by potential lysosomal defects.

## mTOR inhibits autophagy by itself or suppresses TFEB/ACSS2 pathway

mTOR, activated by PI3K/Akt pathway, is a major inhibitor of autophagy. According to the previous reports, mTOR regulates protein synthesis [20] and degradation [21], which demonstrates its significance in tumor progression [22–24]

**Fig. 2:**
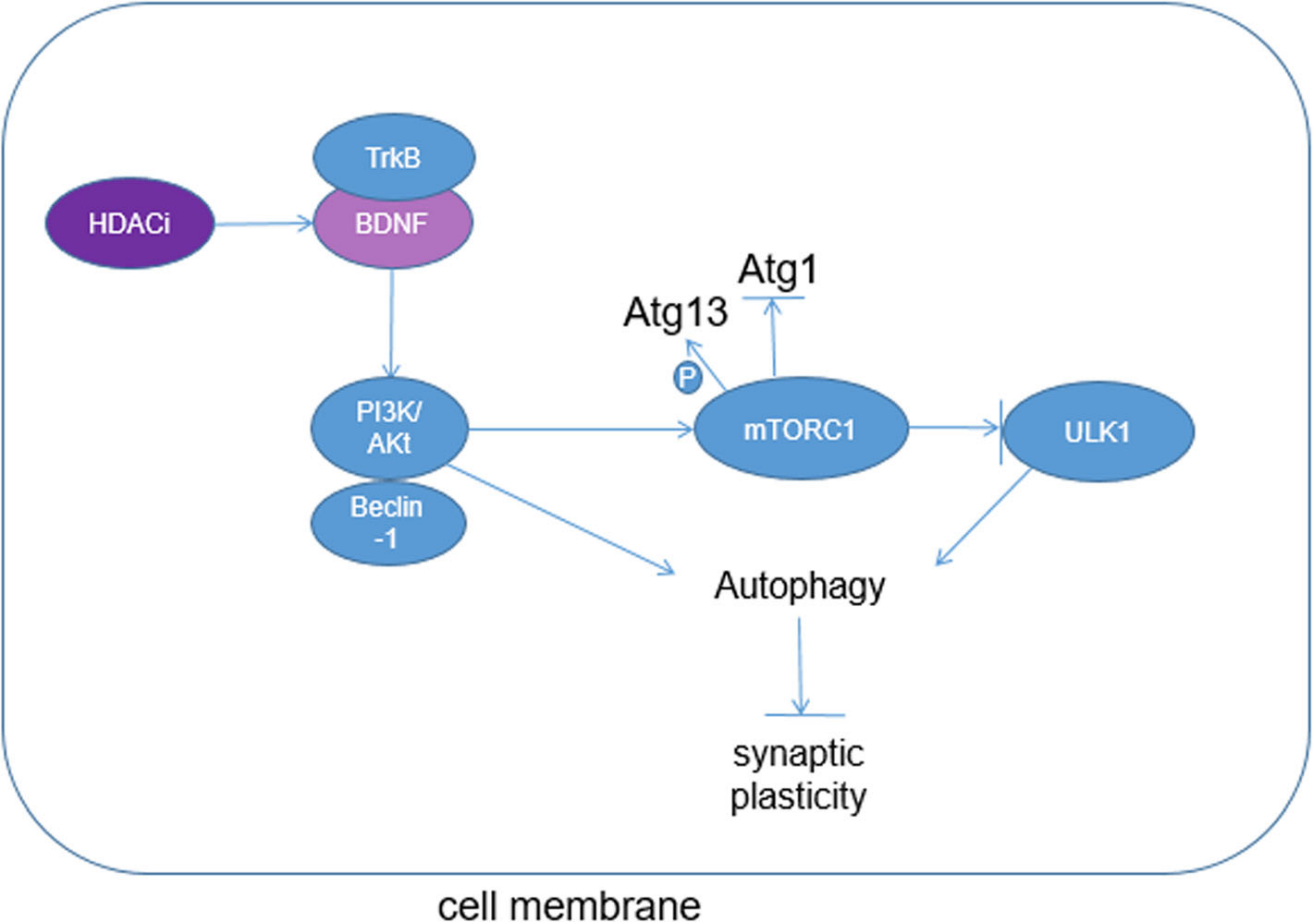
BDNF via TrkB and PI3K/Akt pathway in the hippocampus suppresses autophagic flux and autophagosomes. mTORC1 phosphorylates Atg13 and inhibits Atg1, which is a necessary condition for suppressing autophagy

Some amino acids (AA), such as Leu, Ile, and Val, are known to be potent stimulators of mTOR signaling and protein synthesis in mouse and bovine [25–27]. Other essential AA (EAA), e.g., Lys, His, and Thr, inhibited the mTOR pathway in mammary cells when added at supraphysiological concentrations to AA-depleted cell culture medium [28]. mTOR activity enhances protein synthesis [20] by participating in complex mTORC1. In addition, mTORC1 phosphorylates Atg13 and inhibits Atg1, which is a necessary condition for suppressing autophagy [29] (Fig. 2). Inhibition of mTOR leads to its dissociation from the complex resulting in autophagy induction [30–32]. Insulin or insulin-like growth factor signaling activates mTOR via class-I phosphoinositide-3-kinase (PI3K I) and Akt and, hence, inhibits mammalian autophagy. In the nervous system, mTORC1 activities stimulate the synaptic plasticity and learning of protein synthesis dependent [20, 33, 34]. Rapamycin, an inhibitor of mTOR [35], by inhibiting protein synthesis [36], blocks the axon hyperexcitability and synaptic plasticity of cellular models, hindering learning and memory [37]. Hernandez’s research showed that the mouse, which has complete macroscopic autophagy and mTOR inhibition with rapamycin, can obviously increase the autophagic vesicle (AV) formation in axonal presynaptic terminals, decrease the number of vesicles, and inhibit the release of dopamine that is associated with memory [38, 39]. mTOR alters presynaptic structure and neurotransmission by regulating macroautophagy in presynaptic terminals [39]. These results indicate that mTOR possesses the capability of inhibiting macroautophagy initiation and, therefore, prevents presynaptic terminal from presynaptic structure and neurotransmitter alteration. On the other side, mTOR also prevents TFEB from translocating into nucleus, resulting in the suppression of TFEB/ACSS2 pathway. According to the report, rapamycin (mTOR)-dependent TFEB phosphorylation has the mammalian target at S211 of TFEB [40]. It was triggered by binding TFEB to 14-3-3 proteins, leaving TFEB in the cell cytosol [41], so that it is unable to interact with ACSS2 in the nucleus. Meanwhile, under the influence of energy stress, mTOR activity which is inhibited by the glucose deprivation led to the nuclear translocation and co-localization of TFEB and ACSS2 [14]. However, mTOR inhibition did not affect AMPK activity [42]. This indicates that mTOR suppresses ACSS2-related autophagy by inhibiting nuclear translocation of TFEB, instead of ACSS2.

## ACSS2 suppresses autophagy and enhances memory by upregulating BDNF expression

Brain-derived neurotrophic factor (BDNF) is a protein existing in neurons which can support neuron survival as well as encourage growth and differentiation of neurons. Fernanda [43] showed that an obvious increase of BDNF level in the dorsal hippocampus rather than itself can promote memory consolidation at a later time point. BDNF activates its receptor, tropomyosin receptor kinase B (TrkB), stimulating three distinct intracellular kinase signaling pathway including phospholipase C/protein kinase C, extracellular signal-regulated protein kinase (ERK)/mitogen-activated protein kinase (MAPK), and PI3K/Akt [44–46], which have key functions in autophagy, synaptic plasticity, and memory formation. Vassiliki [47] proved that BDNF via TrkB and PI3K/Akt pathway in the hippocampus suppresses autophagic flux and autophagosomes that are responsible for synaptic defect caused by BDNF deficiency (Fig. 2). BDNF suppresses early steps of phagophore nucleation and elongation transcriptionally, the key components of autophagy. Fasting facilitates memory consolidation by the upregulation of BDNF and inhibition of autophagy in the hippocampus [47]. Autophagy is a crucial component of mediating synaptic plasticity. Tang [48] revealed that autophagy plays an essential role in dendritic spine elimination in the cortex during developmental pruning. A former study [49] showed that post-training trichostain A (TSA)-mediated BDNF increase might correspond with extinction resistance instead of accelerating memory formation. McReynolds [50] found that in dietary restriction condition, ACSS2-CBP mechanism mediates an immediate early gene of activity-regulated cytoskeletal protein (Arc) which is enhanced by basolateral amygdale (BLA) activation. Upregulation of Arc might have access to mediating BDNF influence on synaptic plasticity and memory consolidation [51]. Furthermore, TSA upregulates BDNF and ΔFosB expression and activates CREB [52] as well as increases histone H3 acetylation [53] in the BLA, contributing to the synaptic plasticity and memory consolidation in the hippocampus.

## ACSS2 contributes to HAT by co-localizing with CBP

**Fig. 3:**
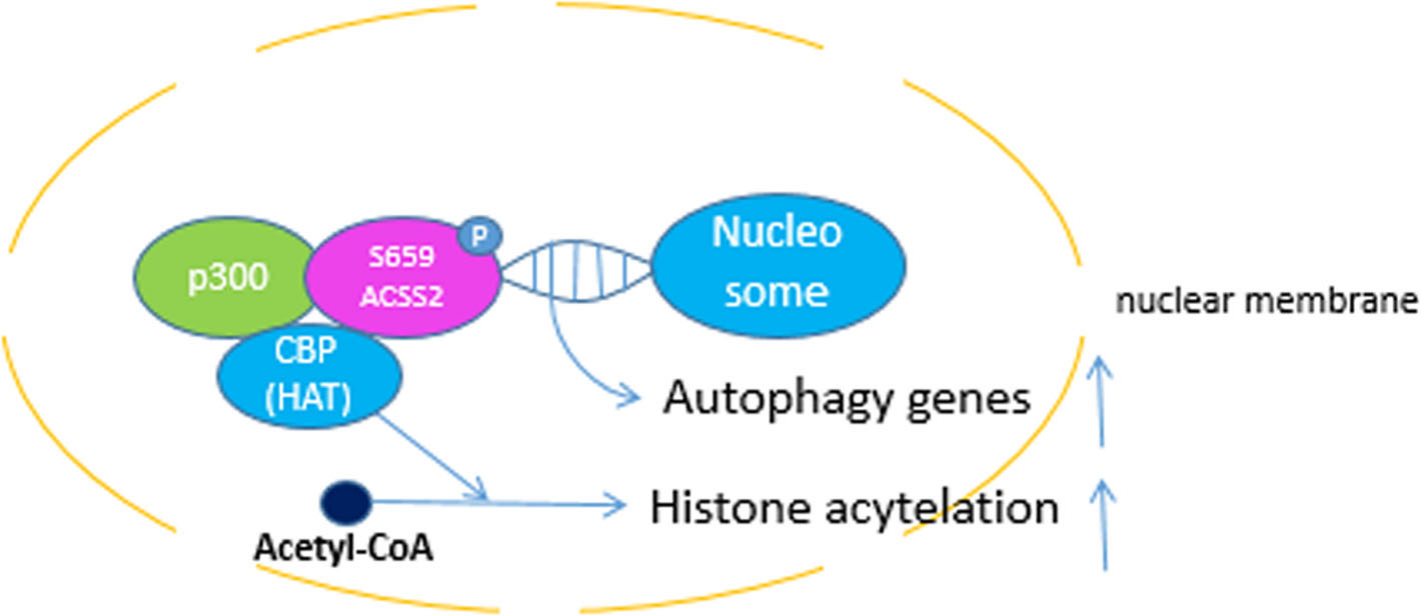
CBP has histone acetyltransferase (HAT) activity in which the metabolite acetyl-CoA is required for histone acetylation. ACSS2 maintains the level of localized histone acetylation by recapturing acetate from histone deacetylation to reform acetyl-CoA, providing substrates for HAT

Hippocampus memory formation and consolidation require the transcription factor (TF) CREB and its coactivator CREB-binding protein (CBP). CBP has histone acetyltransferase (HAT) activity [54] in which the metabolite acetyl-CoA is required for histone acetylation. In Mews’ research [55], immunofluorescence showed that ACSS2 is cytoplasmic in undifferentiated Cath.-a-differentiated (CAD) cells. After ACSS2 translocating into the nucleus of differentiated CAD cells, it co-localized with CBP and E1A-binding protein (p300), one of the TF families corresponding with ACSS2, recruited by neuron-specific motif (Fig. 3). Initial peak analysis [55] showed that the most enriched ACSS2 peaks connect with the strongest histone acetylation enrichment. The most enriched motif which neuronal TF binding is Yin Yang 1 [56] (YY1). YY1 recruits CBP and p300, and ACSS2 activates its HAT’s catalytic activity. ACSS2 maintains the level of localized histone acetylation by recapturing acetate from histone deacetylation to reform acetyl-CoA, providing substrates for HAT. An increased local acetyl-CoA to CoA ratio determines the catalytic activity and substrate specificity of HAT enzymes, giving rise to optimal HAT activity [10, 57, 58]. This finding reveals that histone acetylation can be controlled by altering levels of nuclear acetyl-CoA. Markers, such as H3 lysine 9 acetylation (H3K9ac), H4k4ac, and H4K12ac, were enriched through differentiation at upregulated neuronal genes. Among them, H3K9ac is essential for ACSS2 transcription function [55]. Also, ACSS2 increases the special neuronal gene expression. After ACSS2-CBP mechanism increases histone acetylation locally, immediate early gene transcription is upregulated, including *Egr2*, *Fos*, *Nr2f2*, *Sgk1*, and *Arc*, which play key roles in neuronal plasticity and memory. An increased local acetyl-CoA to CoA ratio ensures optimal acetyltransferase activity. So the level of local histone acetylation can be controlled by mediating the concentration of intracellular acetyl-CoA. Motif analysis [55] reveals that Nrf1, a transcription factor that regulates neurite growth, is predicted binding at 45% of ACSS2 sites, evoking ACSS2-CBP mechanism. However, the specific connection between regulation of Nrf1 and memory consolidation is unclear.

## Dual effect of ACSS2 on memory

Autophagy negatively regulates axon extension [59], so that autophagy may have a negative effect on synaptic plasticity and memory functions. ACSS2 is associated with the expression of autophagy gene. When ACSS2 combines with TFEB in the nucleus, ACSS2 will promote autophagy to impair memory consolidation. However, despite enhancement of autophagy, ACSS2 can also consolidate memory by promoting histone acetylation, which is recognized as a crucial epigenetic process in long-term memory and mice’s memory storage in the dorsal hippocampus [60, 61].

Histone acetylation is manipulated by intracellular acetyl-CoA pool [57]. Under metabolic stress, ACSS2 promotes acetate utilization [61] by recruiting acetate from histone deacetylation, restoring cerebral acetyl-CoA homeostasis. ACSS2 seems to control histone acetylation, even gene expression, by regulating the generation of nuclear acetyl-CoA. A study showed that ACSS2 is primarily a nuclear regulator which upregulates the expression of specific corresponding neuronal genes [55]. These genes, such as immediate early genes, play significant roles in memory regulation. A recent finding [62] supported the hypothesis that ACSS2 is a key mediator between acetate metabolism and neuronal gene regulation through directly binding to chromatin, contributing to hippocampal memory consolidation.

At the same time, a decrease in ACSS2 in the hippocampus can cause downregulation memory-related neuronal genes, affecting long-term potential (LTP). Since LTP of excitatory synaptic transmission is an essential component of the cellular substrates of memory [63, 64], from this aspect, ACSS2 has a potential function in consolidating memory. Recently, a study also found that Nrf1 is critical for the neuronal homeostasis, and lack of Nrf1 results in severe neurodegeneration [65] in mice. We assume that similar results may exist in human neuron cell, and Nrf1 may enhance ACSS2’s function in memory consolidation.

The positive impact of ACSS2-related autophagy on memory seems to be a protective factor because of ACSS2’s function in histone acetylation when neurons are under stress. It seems that ACSS2 could be potentially applied as a novel drug target for neuropsychological disease relief and memory management which encourages us to consider whether we can treat memory disorders by targeting ACSS2 or some related molecules. Therefore, we hypothesize that regulating autophagy of neurons may be a possible way to relieve the pressure of memory impairment, even a potential treatment target for memory diseases. Apparently, the effect of ACSS2 on memory consolidation is both positive and negative, but the integrated effect of ACSS2 on memory is still not fully understood, and more researches in this area are in need.

## Conclusion

Although ACSS2 promotes autophagy which is likely to be a potential risk factor for memory, it simultaneously promotes histone acetylation, an essential process for memory consolidation. Therefore, the effect of ACSS2 on memory consolidation is both positive and negative, yet the overall role of ACSS2 on memory remains uncertain.

## Abbreviations

ACSS2: Acetyl-CoA synthetase 2
AMPK: 5′ AMP-activated protein kinase
Bcl-2: B cell lymphoma 2
BLA: Basolateral amygdale
CaMKKb: Ca2+/calmodulin-dependent protein kinase kinase b
CBP: CREB-binding protein
H3K9ac: H3 lysine 9 acetylation
HAT: Histone acetyltransferase
LKB1: Liver kinase B1
NLS: Nuclear localization signal
PI3K: Phosphatidylinositol 3-kinase
TFEB: Transcription factor EB
TSA: Post-training trichostain A
ULK1: Unc-51-like kinase 1
